# Supplementary material for: Early mucosal events promote distinct mucosal and systemic antibody responses to live attenuated influenza vaccine
Source: Nat Commun. 2023 Dec 5;14:8053. doi: 10.1038/s41467-023-43842-7 (PMC10697962; doi:10.1038/s41467-023-43842-7)
Supplement: Supplementary file 1 — Supplementary Information [file 41467_2023_43842_MOESM1_ESM.pdf]

Supplementary Tables

	**LAIV recipients (n=40)**
Sex (M:F)	9:31
Age (median, range)	22, 19-29
Body Mass Index (median, range)	21.8, 18.0-28.6
Peak nasal inspiratory flow (PNIF; l/min) (median, range)	100, 40-200
Ethnicity	White, n=25 (63%)<br>Asian, n=8 (20%)<br>Mixed, n=4 (10%)<br>Black, n=3 (7%)

Supplementary Table 1 – Demographics of LAIV study participants

Supplementary Figures

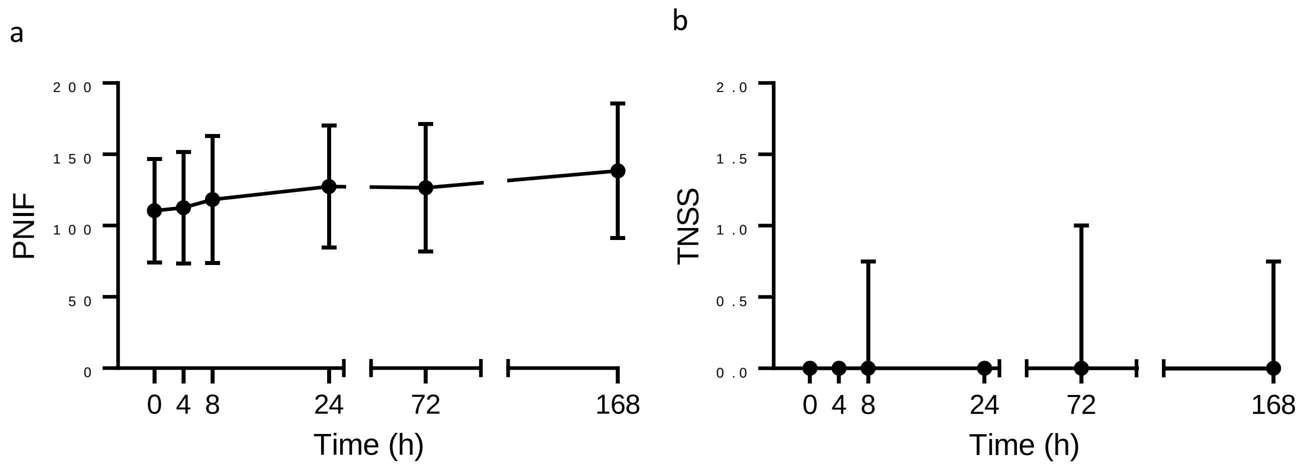

Supplementary Figure 1 – Clinical response to live attenuated influenza vaccination of young adult volunteers

Participants were vaccinated with live attenuated influenza vaccine and symptoms assessed longitudinally. **a** peak nasal inspiratory flow (PNIF) as an objective measure of nasal blockage. **b** Total nasal symptom score (TNSS) as a subjective measure of nasal congestion, sneezing, nasal itching, and rhinorrhea reported by the participant. Figures denote medians and interquartile ranges.

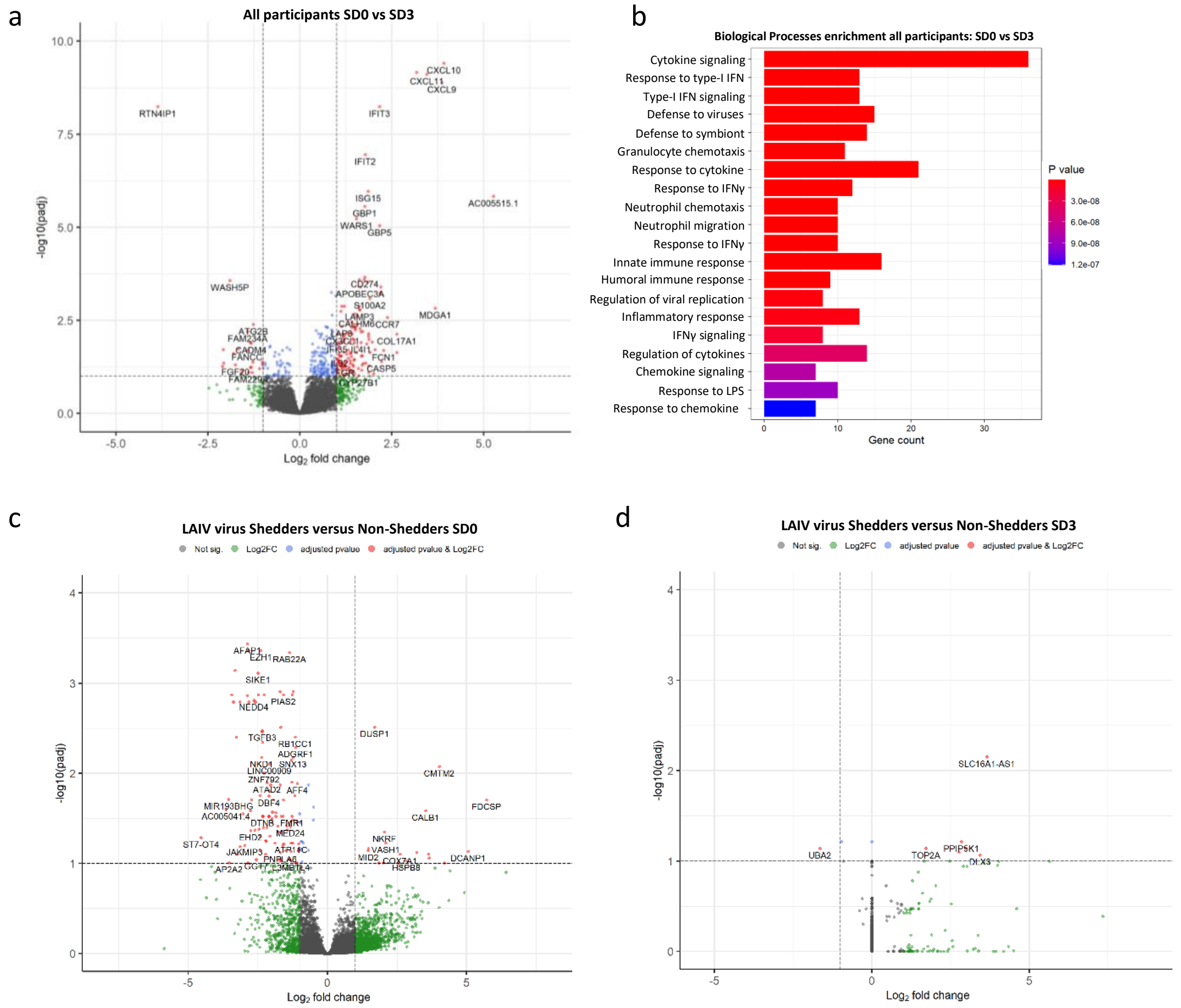

Supplementary Figure 2 – Transcriptional responses to live attenuated influenza virus inoculation in the nose of young adult volunteers

RNA-Sequencing was conducted on nasal tissue samples collected at baseline (SD0, n=34) and at 72 h post-vaccination (SD3, n=26). **a** Volcano plot of differentially expressed genes (DEGs) between SD0 and SD3 of all study participants. **b** Biological processes enriched in DEGs identified in panel A. Study participants were then subcategorised based on whether they shed live attenuated influenza vaccine virus at any time point (“Shedders”, n=20) or not (“Non-shedders”, n=5). **c** DEGs between shedders and non-shedders at SD3. **d** DEGs shedders and non-shedders at SD0. *P*-values in all panels were attained by the Wald test and corrected for multiple testing using the Benjamini and Hochberg method.

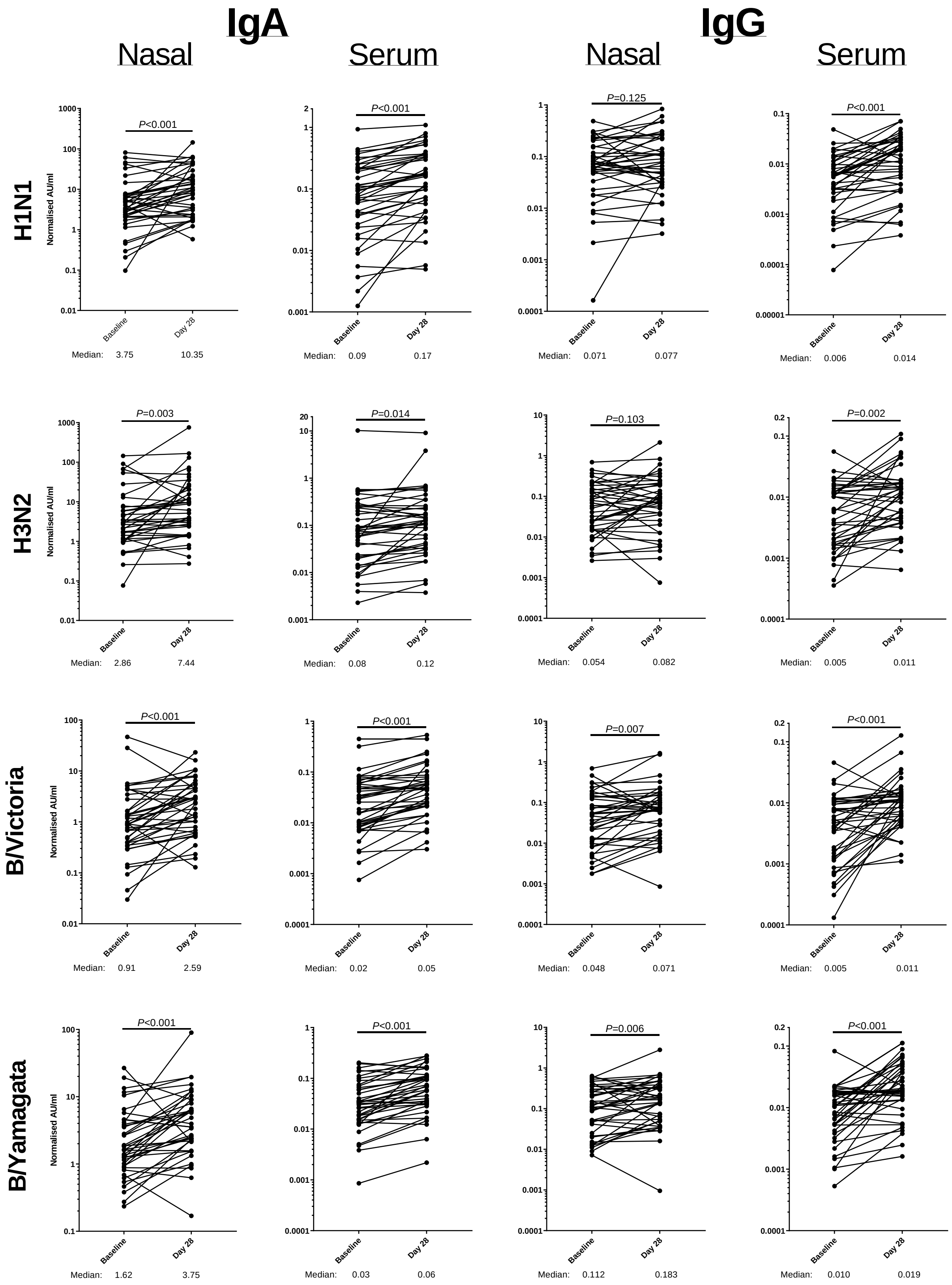
IgA
IgG
Nasal
Serum
Nasal
Serum
H1N1
H3N2
B/Victoria
B/Yamagata
Normalised AU/ml
Baseline
Day 28
P<0.001
Median: 3.75 10.35
P<0.001
Median: 0.09 0.17
P=0.125
Median: 0.071 0.077
P<0.001
Median: 0.006 0.014
P=0.003
Median: 2.86 7.44
P=0.014
Median: 0.08 0.12
P=0.103
Median: 0.054 0.082
P=0.002
Median: 0.005 0.011
P<0.001
Median: 0.91 2.59
P<0.001
Median: 0.02 0.05
P=0.007
Median: 0.048 0.071
P<0.001
Median: 0.005 0.011
P<0.001
Median: 1.62 3.75
P<0.001
Median: 0.03 0.06
P=0.006
Median: 0.112 0.183
P<0.001
Median: 0.010 0.019

Supplementary Figure 3 – Antibody responses to LAIV

IgA and IgG titres against each live attenuated influenza vaccine (LAIV) component haemagglutinin were normalised to total isotype content for each sample type. Statistical significance was tested using Mann-Whitney U tests. All panels represent antibody binding titre data in arbitrary units (AU) normalized to the total level of each isotype of the total LAIV recipient cohort (n=40).

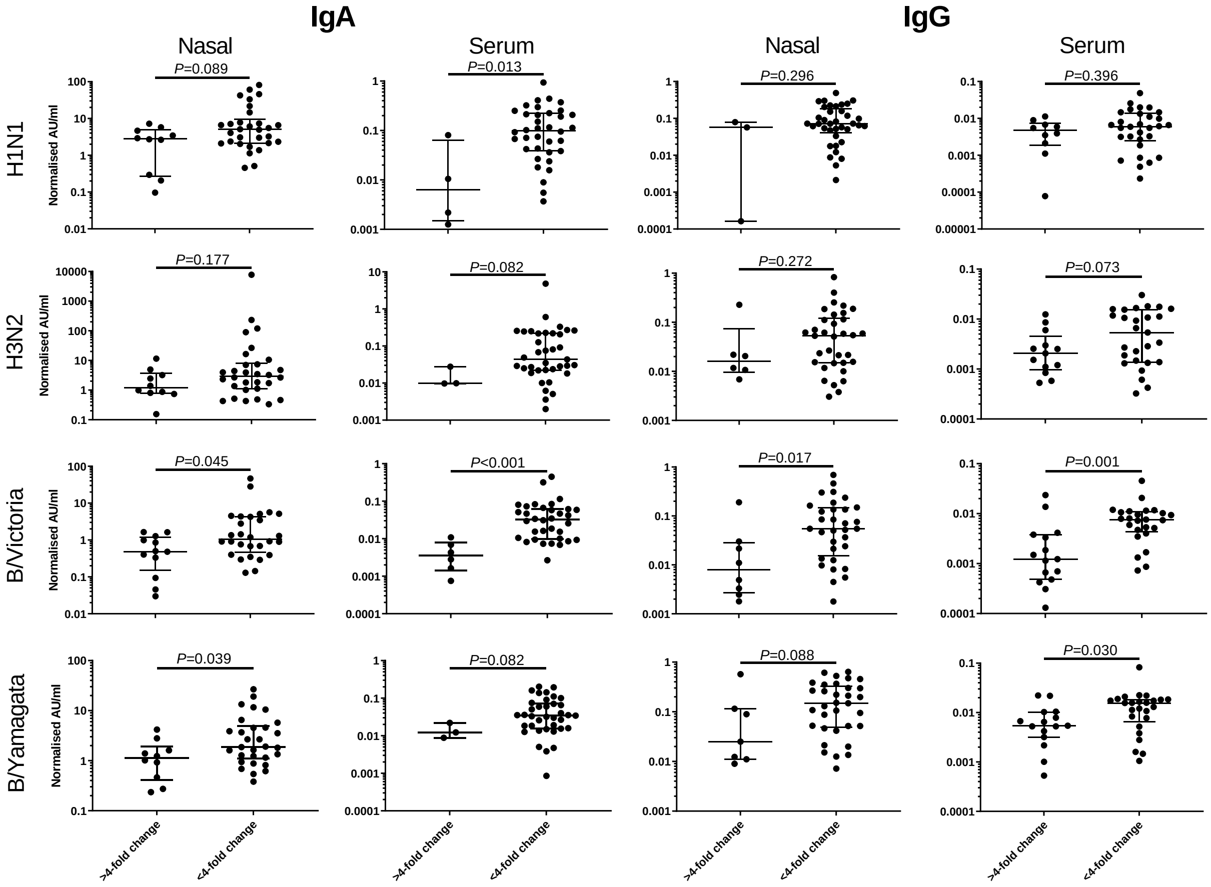

Supplementary Figure 4 – Lower baseline antibody titres in those participants that will seroconvert following LAIV

Normalized antibody titres at baseline (SD0) in those participants that will (">4 fold change") and will not ("<4 fold change") experience seroconversion (defined at ≥4 fold normalized titre rise) at SD28 following LAIV within that sample type/isotype combination. Significance was tested between groups using Mann Whitney tests. All panels represent antibody binding titre data in arbitrary units (AU) normalized to the total level of each isotype of the total LAIV recipient cohort (n=40).

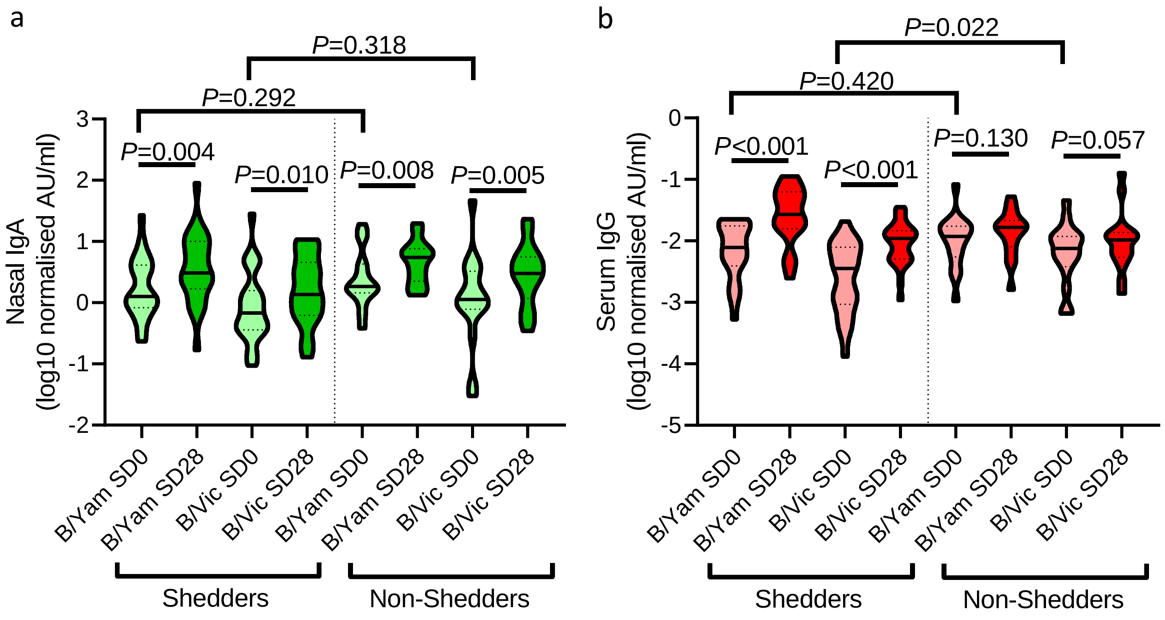

Supplementary Figure 5 – Stronger systemic IgG responses amongst LAIV Shedders

Titres of **a** nasal IgA and **b** serum IgG at baseline (SD0) and day 28 post-inoculation (SD28) with live attenuated influenza vaccine (LAIV) against B/Yamagata ("B/Yam") and B/Victoria ("B/Vic") haemagglutinin antigens. Participants (n=40) are split based on their shedding status for each vaccine virus. Significance was tested between groups using Wilcoxon tests for paired data (between timepoints) and Mann-Whitney tests for unpaired data (between groups). All panels represent antibody binding titre data in arbitrary units (AU) normalized to the total level of each isotype of the total LAIV recipient cohort (n=40). Solid lines represent medians and dotted lines represent interquartile ranges.

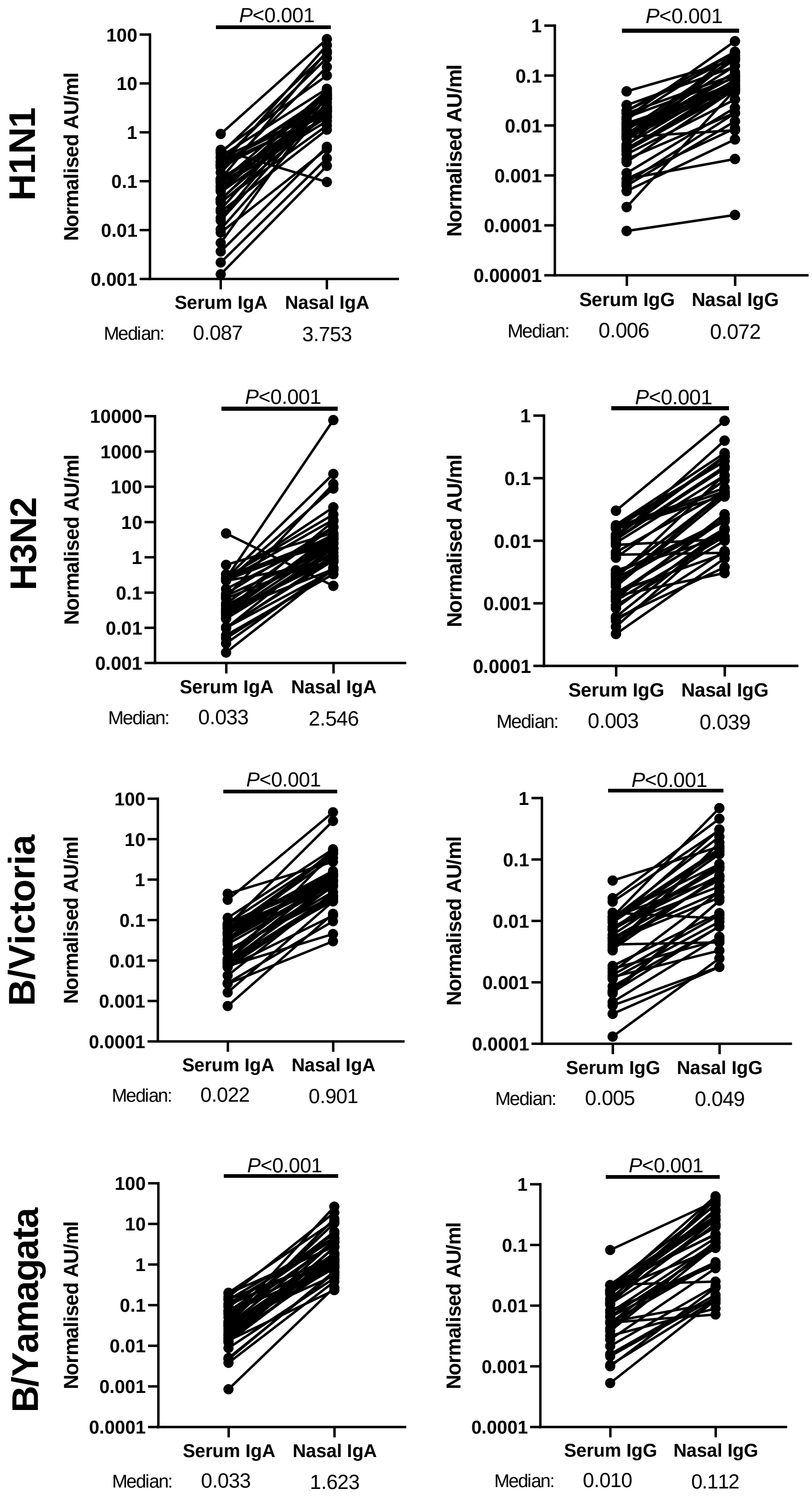
H1N1
H3N2
B/Victoria
B/Yamagata
P<0.001
Normalised AU/ml
Serum IgA
Nasal IgA
Serum IgG
Nasal IgG
Median: 0.087 3.753
Median: 0.006 0.072
Median: 0.033 2.546
Median: 0.003 0.039
Median: 0.022 0.901
Median: 0.005 0.049
Median: 0.033 1.623
Median: 0.010 0.112

Supplementary Figure 6 – Anti-influenza antibodies are disproportionately abundant in the nasal mucosa relative to blood at baseline Normalized antibody titres at SD0 in matched nasal and blood samples (n=40). Significance was tested using Wilcoxon tests for paired data (between sample types within each participant). All panels represent antibody binding titre data in arbitrary units (AU) normalized to the total level of each isotype of the total LAIV recipient cohort (n=40). Lines represent individual paired data.

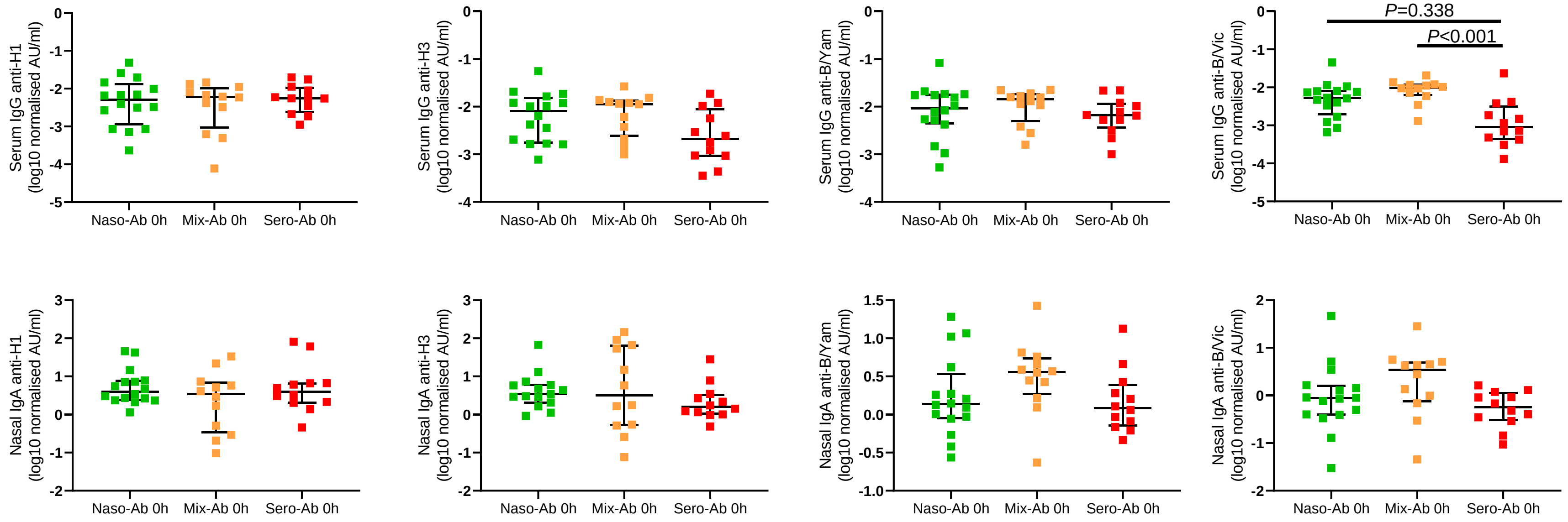

Supplementary Figure 7 – Anti-influenza antibodies at baseline between antibody response type groups Normalized antibody titres at SD0 in nasal and blood samples (n=40). Participants were grouped according to their predominant humoral response; largely nasal IgA ("Naso-Ab", n=16), largely serum IgG ("Sero-Ab", n=12), or a mixed/absent humoral response ("Mix-Ab", n=12). All panels show median and interquartile range values. Significance was tested between groups at baseline (0 h) using Kruskal-Wallis tests with Dunn's correction for multiple comparisons. Lines represent medians and interquartile ranges.

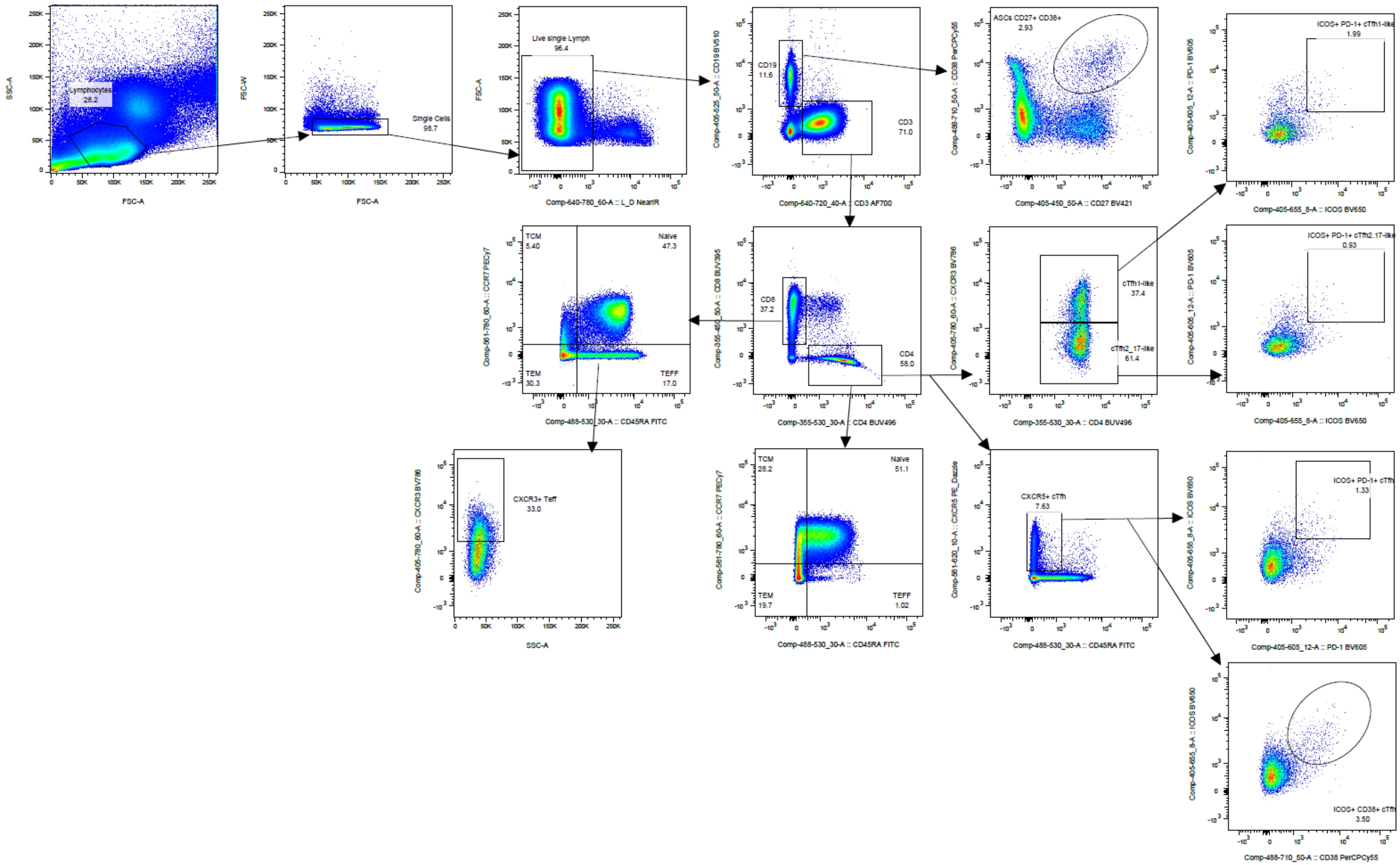

Supplementary Figure 8 – Gating strategies for peripheral blood mononuclear cell populations of interest

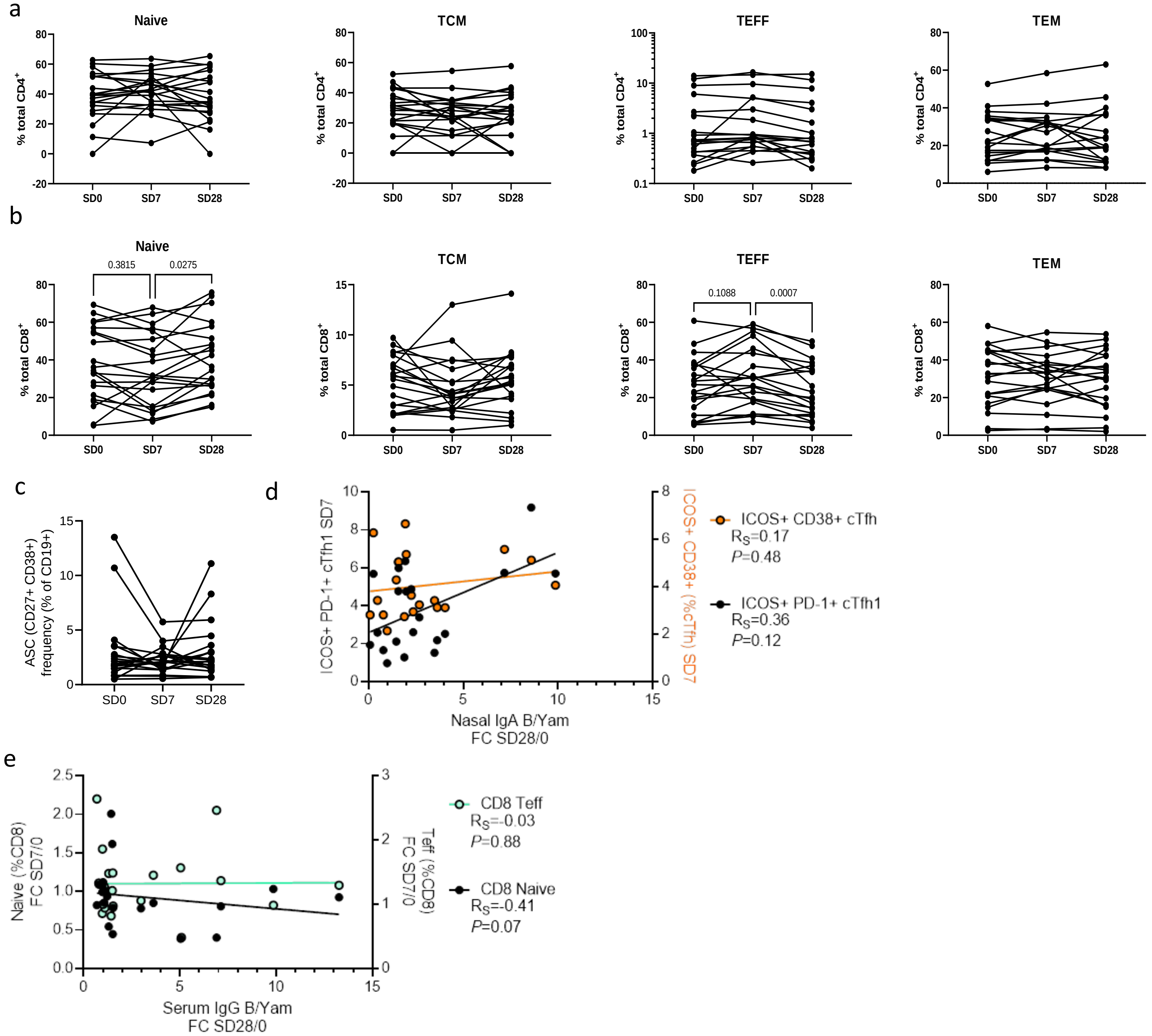

Supplementary Figure 9 – Lymphocyte population frequencies over time

T cells were subgrouped into naïve ($CD45RA^+$ $CCR7^+$), T central memory (TCM; $CD45RA^-$ $CCR7^+$), T effector (TEFF; $CD45RA^+$ $CCR7^-$), and T effector memory (TEM; $CD45RA^-$ $CCR7^-$) phenotypes for **a** $CD4^+$ T cells and **b** $CD8^+$ T cells from participants (n=23) at SD0, 7 and 28. **c** Antibody secreting cells (ASC) frequency as a % of total $CD19^+$ B cells. **d** Correlation between nasal IgA anti-B/Yamagata titre fold-changes between SD28 and SD0 versus cTfh1-like cell activation ($ICOS^+$ $PD\text{-}1^+$) and alternatively activated ($ICOS^+$ $CD38^+$) total cTfh at SD7. **e** Correlation between serum IgG anti-B/Yamagata titre fold-changes between SD28 and SD0 versus naïve and T_{EFF} $CD8^+$ T cell fold-changes at SD7 relative to SD0. Statistical significance in panels a-c was assessed by Mann-Whitney U tests and significant or trending *P* values are displayed. Panels d and e utilised Spearman's correlations for non-parametrically distributed data.
