# Supplementary material for: Early mucosal events promote distinct mucosal and systemic antibody responses to live attenuated influenza vaccine
Source: Nat Commun. 2023 Dec 5;14:8053. doi: 10.1038/s41467-023-43842-7 (PMC10697962; doi:10.1038/s41467-023-43842-7)
Supplement: Supplementary file 3 — Reporting Summary [file 41467_2023_43842_MOESM3_ESM.pdf]

nature portfolio

Corresponding author(s): Ryan Stuart Thwaites

Last updated by author(s): Oct 23, 2023

Reporting Summary

Nature Portfolio wishes to improve the reproducibility of the work that we publish. This form provides structure for consistency and transparency in reporting. For further information on Nature Portfolio policies, see our Editorial Policies and the Editorial Policy Checklist.

Statistics

For all statistical analyses, confirm that the following items are present in the figure legend, table legend, main text, or Methods section.

n/a	Confirmed	
☐	☒	The exact sample size (*n*) for each experimental group/condition, given as a discrete number and unit of measurement
☐	☒	A statement on whether measurements were taken from distinct samples or whether the same sample was measured repeatedly
☐	☒	The statistical test(s) used AND whether they are one- or two-sided<br>*Only common tests should be described solely by name; describe more complex techniques in the Methods section.*
☐	☒	A description of all covariates tested
☐	☒	A description of any assumptions or corrections, such as tests of normality and adjustment for multiple comparisons
☐	☒	A full description of the statistical parameters including central tendency (e.g. means) or other basic estimates (e.g. regression coefficient) AND variation (e.g. standard deviation) or associated estimates of uncertainty (e.g. confidence intervals)
☐	☒	For null hypothesis testing, the test statistic (e.g. *F*, *t*, *r*) with confidence intervals, effect sizes, degrees of freedom and *P* value noted<br>*Give P values as exact values whenever suitable.*
☒	☐	For Bayesian analysis, information on the choice of priors and Markov chain Monte Carlo settings
☒	☐	For hierarchical and complex designs, identification of the appropriate level for tests and full reporting of outcomes
☐	☒	Estimates of effect sizes (e.g. Cohen's *d*, Pearson's *r*), indicating how they were calculated

Our web collection on statistics for biologists contains articles on many of the points above.

Software and code

Policy information about availability of computer code

Data collection	Data collection used commercially available software: Mesoscale Discovery Workbench v4.0, BD Diva v6.1.3, Bio-Plex Manager v6.0 , and Microsoft Excel (v2306)
Data analysis	Data analysis utilised GraphPad Prism v9 or R v4.2.1. Correlation matrices used GraphPad Prism or R packages ggplot2 and ggcorrplot. Heatmaps used the R package ComplexHeatmap.<br>Sequencing quality control was performed using FastQC (v0.11.7), samtools stats (v1.9) and Qualimap (v2.2.2c). MultiQC(1.9) was used to summarize and asses the libraries QC metrics for sample selection. Fastp (v 0.20.1) was used to trim the adapters and reads were mapped against Ensembl 99 and quantified using the Salmon tool. Nextflow workflow and Bioconda software management tool was used to run the workflow. DeSEQ2 (v 1.34.0) was used to normalize the counts and to identify differentially expressed genes.volcano plots were generated using R package EnhancedVolcano R package (v 1.8.0) and ggplot2 (v 3.3.5). For gene ontology analysis the list of DEGs obtained from DeSEQ1 were applied as input and performed using EnrichR “GO Biological Process”.

For manuscripts utilizing custom algorithms or software that are central to the research but not yet described in published literature, software must be made available to editors and reviewers. We strongly encourage code deposition in a community repository (e.g. GitHub). See the Nature Portfolio guidelines for submitting code & software for further information.

Data

Policy information about availability of data

All manuscripts must include a data availability statement. This statement should provide the following information, where applicable:

- Accession codes, unique identifiers, or web links for publicly available datasets
- A description of any restrictions on data availability
- For clinical datasets or third party data, please ensure that the statement adheres to our policy

Transcriptomics data were deposited in GEO with the accession identifier GSE230494 and are publicly available at (https://www.ncbi.nlm.nih.gov/geo/query/acc.cgi?acc=GSE230494). The reference genome used (GRCh38) is available publicly available through the National Center for Biotechnology Information RefSeq assembly GCF_000001405.40 (https://www.ncbi.nlm.nih.gov/datasets/genome/GCF_000001405.40/). The remaining data that support the findings of this study are available from the corresponding authors upon reasonable request.

Research involving human participants, their data, or biological material

Policy information about studies with human participants or human data. See also policy information about sex, gender (identity/presentation), and sexual orientation and race, ethnicity and racism.

Reporting on sex and gender	Self-reported sex is detailed in the participant recruitment and demographics descriptions. The study group is not disaggregated on the basis of sex, as the study design was not powered for subgroup analysis on the basis of sex. The frequency of males and females in the study group is reported in the manuscript.
Reporting on race, ethnicity, or other socially relevant groupings	Self-reported race and ethnicity data were collected using a standardised NHS self-reporting tool. These data are not reported in the manuscript as no sub-set analysis on the basis of race or ethnicity is performed.
Population characteristics	Inclusion was restricted to 18-30 year olds to meet the study design. Exclusion criteria were age <18 years or >30 years, receipt of an influenza vaccine in the last 2 years, egg allergies, current smoking, pregnancy, use of any medication that may affect the immune system, current acute illness (including respiratory infections), clinically diagnosed influenza in the last 2 years, any long-term health problem (including asthma and other conditions that would trigger a recommendation for influenza immunization), history of Guillain-Barre syndrome, receipt of any vaccine in the past 4 weeks, and living with an immunocompromised person.
Recruitment	Participants were recruited by local poster advertisement. Inclusion was restricted to 18-30 year olds to meet the study design.
Ethics oversight	The study was approved by the London Camberwell St Giles Research Ethics Committee (REC, reference 18/LO/0904) and the Health Research Authority. The study was registered on clinicaltrials.gov (NCT04110366).

Note that full information on the approval of the study protocol must also be provided in the manuscript.

Field-specific reporting

Please select the one below that is the best fit for your research. If you are not sure, read the appropriate sections before making your selection.

[x] Life sciences [ ] Behavioural & social sciences [ ] Ecological, evolutionary & environmental sciences

For a reference copy of the document with all sections, see nature.com/documents/nr-reporting-summary-flat.pdf

Life sciences study design

All studies must disclose on these points even when the disclosure is negative.

Sample size	Power calculations were performed based on the trial endpoints for humoral immune responses in both the mucosa (primary endpoint, for which no comparable data existed) and blood (secondary endpoint, for which comparable data did exist. Power calculations therefore considered published blood HAI response data. A group size of n=40 was chosen on the basis of previous reported indicating a 50-75% rate of LAIV vaccine virus HAI seroconversion amongst recipients in this age category (Ohmit et al., Journal of Infectious Disease, Volume 204, Issue 12, 2011). On this basis we anticipated n=20-30 participants to have serum HAI responses, and n=10-20 to not have detectable serum antibody responses to LAIV. Powering analysis demonstrated that such numbers of participants per serum response or no-serum response arms would yield ample power (>99%) to observe significant antibody (HAI) induction between baselines and SD28 where sero-responses resulted in a HAI change of >4-fold titre increase.
Data exclusions	No data were excluded from the publication. The n of samples analysed by flow cytometry (n=23) is smaller than the full study arm (n=40) for two reasons: i) In some cases insufficient PBMCs were available from all three timepoints of a volunteer, in such cases no samples were analyseed, ii) the -200C freezer in which PBMC were retained failed late in the study course, rendering some samples (and additional PBMC aliquots) unusable.
Replication	Experiments were conducted on samples arising from a single cohort (n=40) of volunteers. No replication cohort was recruited by design. Experimental results were replicated in duplicate, with a mean of duplicates taken if CV<15%. If CV>15%, sample analysis was repeated. All attempts at experimental replication were successful.

Randomization	Randomisation to the placebo or LAIV arms was not conducted as the placebo arm was, by design, a shorter duration (seeking only to inform analyses of early nasal mediator responses). The shedding/no-shedding status/grouping of participants was not known to the participants or investigators at the time of the clinical study.
Blinding	Blinding was not performed as the study did not include an equivalent placebo control arm by design, but rather sought to understand the drivers of variability in biological responses to vaccination.

Reporting for specific materials, systems and methods

We require information from authors about some types of materials, experimental systems and methods used in many studies. Here, indicate whether each material, system or method listed is relevant to your study. If you are not sure if a list item applies to your research, read the appropriate section before selecting a response.

Materials & experimental systems

n/a	Involved in the study	
☐	☒	Antibodies
☒	☐	Eukaryotic cell lines
☒	☐	Palaeontology and archaeology
☒	☐	Animals and other organisms
☐	☒	Clinical data
☒	☐	Dual use research of concern
☒	☐	Plants

Methods

n/a	Involved in the study	
☒	☐	ChIP-seq
☐	☒	Flow cytometry
☒	☐	MRI-based neuroimaging

Antibodies

Antibodies used	Antibodies from BD: anti-CD4 (clone SK3, BUV496, Cat#564651, Lot#6259653, 1:20), -CD8 (clone RPA-T8, BUV395, Cat#563795, Lot#7069910, 1:40), and -CD278 (Inducible T-cell COStimulator, ICOS; clone DX29, BV650, Cat#563832, Lot#7198562, 1:20)<br>All remaining antibodies were from Biolegend: anti-CD19 (clone HIB19, BV510, Cat#302242, Lot#B239285, 1:20), -CD279 (Programmed cell death protein 1, PD-1; clone EH12.2H7, BV605, Cat#329924, lot#B238715, 1:20), -CCR7 (clone G043-H7, PE/Cy7, Cat#353226, Lot#B238508, 1:20), -CD38 (clone HB-7, PerCPCy5.5, Cat#356613, Lot#B239525, 1:40), -CD32 (clone FUN-2, PE, Cat#303205, Lot#B211165, 1:80), -CD45RA (clone HI100, FITC, Cat#304106, Lot#B202186, 1:40), -CD27 (clone M-T271, BV421, Cat#356417, Lot#B243455, 1:40), -CD127 (clone A019D5, BV711, Cat#351327, Lot#B228247, 1:40), -CXCR3 (clone G025H7, BV786, Cat#353737, Lot#B236359, 1:40), -CD3 (clone OKT3, AF700, Cat#353737, Lot#B223632, 1:80), -CXCR5 (clone J25D4, PE/Dazzle, Cat#356928, Lot#B226046, 1:80)
Validation	Antibody panel staining on human PBMC was validated using fluorescence minus one (FMO) optimisation and QC prior to and throughout the study. The panel design and optimisation are previously reported as detailed in the manuscript, in the publication DOI 10.1038/s41598-019-51961-9

Clinical data

Policy information about clinical studies
All manuscripts should comply with the ICMJEguidelines for publication of clinical research and a completedCONSORT checklist must be included with all submissions.

Clinical trial registration	clinicaltrials.gov (NCT04110366)
Study protocol	clinicaltrials.gov (NCT04110366), full protocol available at: https://classic.clinicaltrials.gov/ProvidedDocs/66/NCT04110366/Prot_SAP_000.pdf
Data collection	Data were collected per protocol within the Imperial Clinical Respiratory Research Unit (ICRRU) at St Mary's Hospital, Imperial College London, between June 2018 and July 2022. All participant records and data were handled within the ICRRU. Analysis of participant samples was conducted at the Medical School Building, St Marys Hospital, Imperial College London, with the exception of HAI assays and viral load quantification, which were conducted by Public Health England (now UK Health Security Agency), London.
Outcomes	Primary and secondary endpoints, defined in the study protocol were assessed as follows: Humoral binding titre assays were analysed using custom multiplex immunoassays donated by AstraZeneca for the quantification of antbody titres between baseline and SD28 samples. HAI assays and viral load quantification of viral shedding were conducted at Public Health England (now UK Health Security Agency), London to meet secondary endpoints.

Flow Cytometry

Plots

Confirm that:

- [x] The axis labels state the marker and fluorochrome used (e.g. CD4-FITC).
- [x] The axis scales are clearly visible. Include numbers along axes only for bottom left plot of group (a 'group' is an analysis of identical markers).
- [x] All plots are contour plots with outliers or pseudocolor plots.
- [x] A numerical value for number of cells or percentage (with statistics) is provided.

Methodology

Sample preparation	Peripheral blood mononuclear cells were isolated using histopaque gradients and frozen ahead of cytometry.
Instrument	All flow cytometry data acquisition was performed on a single BD Fortessa that was underwent QC validation using CST beads and compensation prior to each analytical run.
Software	Flow cytometry data acquisition used BD Diva software. All flow cytometry data analysis was conducted using FlowJo v10.
Cell population abundance	No cell sorting was performed in this study. Population frequencies are reported throughout the manuscript. A minimum of 1x10^6 events were recorded in every flow cytometry sample.
Gating strategy	The gating strategy is detailed in the manuscript supplementary materials. Briefly, lymphocytes were gated of SSC and FSC, followed by single cells (based on FSC-A and FSC-W). Live cells were then gated based on live/dead staining (NearIR). CD19 and CD3 were next used to identify B cells and T cells, respectively. Of the CD19+ B cells, CD27 and CD38 were then used to identify antibody secreting cells.<br>For the CD3+ T cells, CD4 and CD8 T cells were then distinguished. T cell phenotype was determined based on CD45RA and CCR7. CD8+ CD45RA+ CCR7- T effector cells was further categorised as CXCR3+/-. Of the CD4s, additional gating identified CXCR5+ cTfh. These were further subsetted on ICO and PD-1, and ICOS and CD38.

- [x] Tick this box to confirm that a figure exemplifying the gating strategy is provided in the Supplementary Information.
